# Estimation of the diameter and cross-sectional area of the internal jugular veins in adult patients

**DOI:** 10.1186/cc8200

**Published:** 2009-12-09

**Authors:** Déborah Tartière, Philippe Seguin, Charlotte Juhel, Bruno Laviolle, Yannick Mallédant

**Affiliations:** 1 Service d'Anesthésie-Réanimation 1, Université de Rennes 1, Hôpital Pontchaillou, 2 rue Henri Le Guilloux, 35000 Rennes, France; 2 Département d'Imagerie Médicale, Université de Rennes 1, Hôpital Pontchaillou, 2 rue Henri Le Guilloux, 35000 Rennes, France; 3 Laboratoire de Pharmacologie - CIC Inserm 0203, Université de Rennes 1, Hôpital Pontchaillou, 2 rue Henri Le Guilloux, 35000 Rennes, France; 4 Service d'Anesthésie-Réanimation 1, Université de Rennes 1, Inserm U620, Hôpital Pontchaillou, 2 rue Henri Le Guilloux, 35000 Rennes, France

## Abstract

**Introduction:**

Unawareness of an asymmetry between the right and left internal jugular vein (IJV) and methodological pitfalls in previous studies raise concerns about such asymmetry. Hence the aim of this prospective non-interventional study was to validate the hypothesis that right IJV diameter is greater than those of left IJV and to determine the cross-sectional area of the IJVs using computed tomography (CT)-scans and original automatic software.

**Methods:**

All consecutive adult outpatients who underwent a thoracic contrast-enhanced (TCE) helical CT-scan during a 5-month period were included. To determine diameter and cross sectional area of the IJVs, we used Advanced Vessel Analysis software integrated in a CT-scan (Advanced Vessel Analysis on Advantage Workstation Windows 4.2; General Electrics) allowing automatic segmentation of vessels and calculation of their diameters and cross-sectional areas.

**Results:**

A total of 360 TCE CT-scans was performed; 170 were excluded from the analysis. On the remaining 190 CT scans, the diameter and cross-sectional area of the right IJV were significantly greater than those of the left IJV (17 ± 5 mm [median: 17 mm, range: 13 to 20 mm] *vs*. 14 ± 5 mm [median: 13 mm, range: 10 to 16 mm], P < 0.001; and 181 ± 111 mm^2 ^[median: 160 mm^2^, range: 108 to 235 mm^2^] *vs*. 120 ± 81 mm^2 ^[median: 102 mm^2^, range: 63 to 168 mm^2^], P < 0.001, respectively).

**Conclusions:**

In a general population of adult outpatients, the diameter and cross-sectional area of the right IJV were significantly greater than those of the left IJV. This could be an additional argument to prefer right over left IJV cannulation.

## Introduction

When central intravenous access is required and internal jugular vein (IJV) puncture is chosen, the right-sided vein is generally preferred because it provides a direct pathway to the superior vena cava and avoids thoracic duct injury [1]. Moreover, left IJV cannulation is deemed to be more difficult, carrying a higher rate of complications [2]. Finally, it has been reported that the left IJV is smaller than the right. Lobato and colleagues showed that the cross-sectional area of the right IJV was greater than that of the left IJV in the majority (80%) of 50 healthy volunteers [3]. Such asymmetry has been confirmed in intensive care patients, where the right IJV was equal or dominant in 62.5% of patients [4]. Nevertheless, in these two ultrasound studies, the precise diameter and cross-sectional area of the right and left IJV were not reported. Moreover, some limitations of the ultrasound techniques, such as excessive vessel pressure by the probe, neck positioning, and no visualization of the brachiocephalic vein, superior vena cava and mediastinum, could lead to inaccurate measurements. Anatomical variations of the IJVs and their relation to the common carotid artery were evaluated in a retrospective study of 88 patients using computed tomography (CT) scans. The right IJV was usually larger (79.5%) than the left: the mean diameters of the right and left IJVs were 14.10 mm and 11.74 mm, respectively [5]. However, the cross-sectional area was not measured and no statistical analysis was performed in this study. Moreover, it appeared that the IJVs are oblong rather than round [5].

It is noteworthy that in a recent review on central venous catheterization, the difference in size of the right and left IJV was not mentioned when the advantage of right over left IJV cannulation was discussed [1].

The aim of this prospective study was to validate the hypothesis that the diameter of the right IJV is greater than the left one, and to determine cross-sectional areas of both IJVs using CT scans and original automatic software.

## Materials and methods

This observational study was carried out between 1 March and 31 July 2008 in the University Hospital of Rennes, France. The local ethics committee waived the need for informed consent.

The maximal diameter and cross-sectional area of the left and right IJV were analyzed prospectively in all consecutive outpatients who underwent a thoracic contrast-enhanced (TCE) helical CT-scan (Multidetector GE Lightspeed; GE Healthcare Medical Systems, Milwaukee, WI, USA) during the study period.

The primary goal of this study was to validate the hypothesis that the diameter of the right IJV is greater than the left one, and secondary to determine the cross-sectional area of both IJVs.

**Figure 1:**
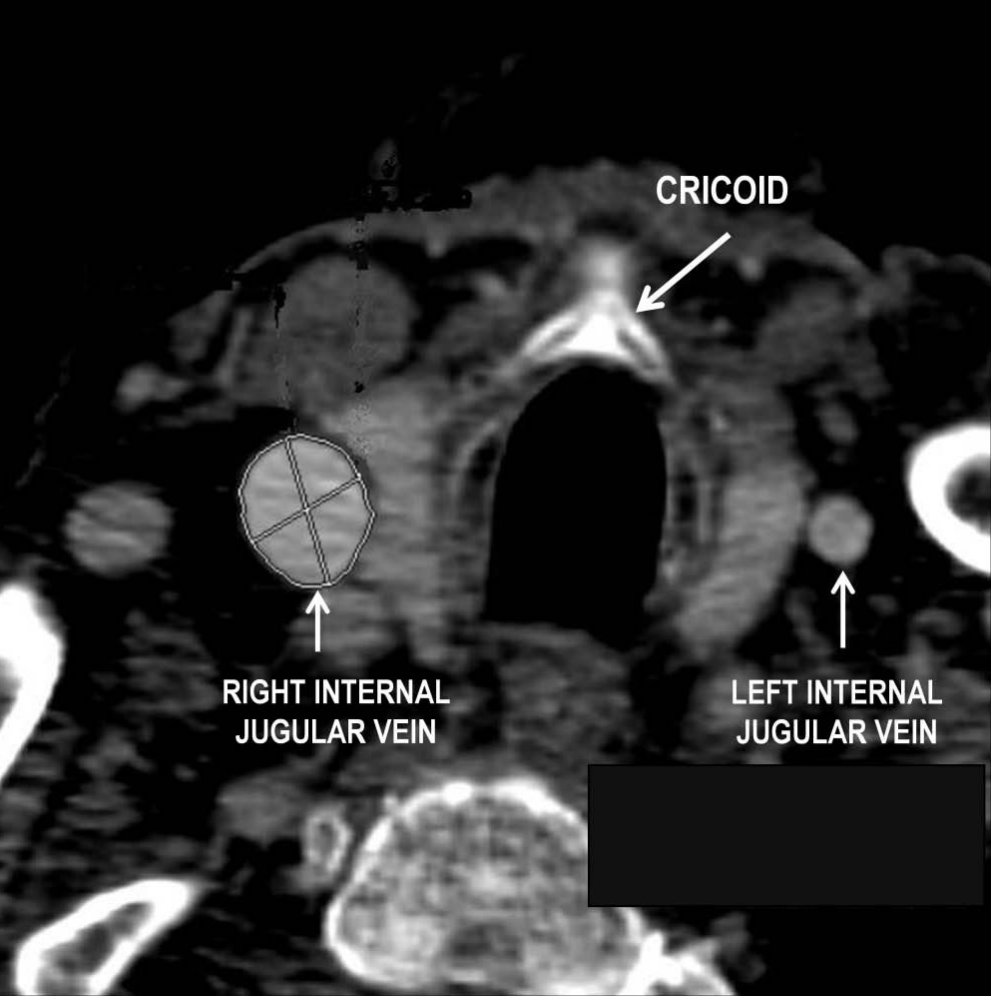
Example of a thoracic contrast-enhanced helical computed tomography scan obtained from a patient with a large right internal jugular vein.

The CT scan examination was standardized. Briefly, all patients were in the supine position, with a neutral head position, breathing spontaneously, and the acquisition protocol was as follows: breath-holding acquisition for 50 seconds after intravenous injection of 120 ml iodinated contrast agent; flow rate: 3 ml/s; 300 slices; slice thickness: 1.25 mm/1.2 mm interval. Thereafter, the diameter and cross-sectional area of the left and right IJVs were measured at the level of the cricoid cartilage (Figure 1). The level of the cricoid cartilage was chosen because this corresponds to the central approach (apex of the triangle formed by the medial and lateral portions of the sternocleidomastoid muscle and clavicle) and anterior approach (at the level of the cricoid cartilage along the medial edge of the sternocleidomastoid muscle) for venous puncture [6]. These measurements were made automatically with Advanced Vessel Analysis software provided with the CT scan workstation (Advanced Vessel Analysis on ADW 4.2; General Electrics, Chalfont St Giles, UK) allowing automated segmentation of vessels and calculation of their diameters and cross-sectional areas. Intraobserver and interobserver correlations for automated 3D CT angiography analysis method have been previously evaluated both for diameter and area, and were 0.89 and 0.90, and 0.90 and 0.91, respectively [7]. The IJV is usually elliptical and only the maximal diameter is reported.

The advantage of these approaches is to have a lateral position to the common carotid artery and a lower risk of arterial puncture. In addition, most anatomical studies on the IJVs have focused on this level [2,3,5,7-9].

The patients' age, sex, and indication for CT scan were recorded. The thoracic CT scans and the maximal diameter and cross-sectional area of the IJVs were measured and analyzed by the same investigator (CJ). All CT scans were examined carefully to exclude any cases that could interfere with IJV measurements. Notably, the absence of extraluminal compression of the vessels was verified as the absence of mediastinum processes.

### Sample size and statistical analysis

Our primary hypothesis was that the diameter was larger in the right IJV than in the left IJV. In a previous unpublished retrospective study performed of CT scans from the radiology department of our hospital, we determined that the mean diameter of right IJV was 18 ± 11 mm. In this context, at least 177 patients were required to detect a difference of 3 mm between the two IJV diameters with a 5% type I error rate and a power of 90% (nQuery Advisor, Statistical Solutions Ltd, Cork, Ireland).

All statistical analyses were performed using SAS v 9.1.3 software (SAS Institute, Cary, NC, USA). Data are expressed as mean ± standard deviation unless otherwise noted. Mean values of the right and left IJV diameters and cross-sectional areas were compared using the paired Student's t-test. For other analysis, the Student's t-test or Wilcoxon rank sum test was used as appropriate. For all analyses, a *P *value less than 0.05 was considered statistically significant.

## Results

A total of 360 TCE CT scans were performed during the study period; 170 were excluded from the analysis (problem with radio-contrast injection (n = 54), inappropriate CT scan sections (n = 108), internal jugular and subclavian catheters previously in place (n = 3), superior vena cava thrombosis (n = 1), no visible left IJV (n = 1), right pneumonectomy (n = 1), compressive mediastinal mass (n = 1), thyroidectomy (n = 1)). Accordingly, a total of 190 CT scans was analyzed.

The 190 patients included 132 men and 58 women (age 60 ± 15 years, range: 18 to 93 years; Table 1). The indications for TCE CT scans were as follows: staging and follow up of cancer (n = 104), post-surgical controls (n = 30), abdominal disease (n = 19), cardiac or respiratory problems (n = 8), inflammatory process (n = 6), sepsis (n = 6), lymphadenopathies or various chest radiograph abnormalities (n = 6), and other (n = 11).

**Table 1:** Demographic data, and diameter and cross-sectional area of the right and left IJV

**Age, mean ± SD (range)**	60 ± 15 (18-93)	
**Sex, M/F, n**	132/58	
	
	**Right IJV**	**Left IJV**
**Diameter (mm)**	17 (13-20)	13 (10-16)
**Cross-sectional area (mm^2^)**	160 (108-236)	102 (63-168)

Data are expressed as median (range) unless otherwise stated.

F = female; IJV = internal jugular vein; M = male; SD = standard deviation.

The diameter and cross-sectional area of the right IJV were significantly greater than those of the left (17 ± 5 mm (median: 17 mm, range: 13 to 20 mm) *vs*. 14 ± 5 mm (median: 13 mm, range: 10 to 16 mm), *P *< 0.001; and 181 ± 111 mm^2 ^(median: 160 mm^2^, range: 108 to 236 mm^2^) *vs*. 120 ± 81 mm^2 ^(median: 102 mm^2^, range: 63 to 168 mm^2^), *P *< 0.001, respectively; Table 1). Right IJV diameter was equal or superior to left IJV diameter, ± 1 mm and ± 2 mm, in 75% and 80%, respectively. Right IJV cross-sectional area was greater than left IJV area in 71% of patients and was 1.5- and 2.0-times in 54% and 34% of cases, respectively. Diameter and cross-sectional area of the right and left IJV were not significantly different according to age and sex (data not shown).

## Discussion

This study shows that in a general population of adult patients the diameter and cross-sectional area of the right IJV were significantly greater than those of the left IJV. This is an argument for right over left IJV cannulation.

For many years, physicians have looked for ways of increasing the success rate and reducing the complications and morbidity associated with central venous line placement. With regard to IJV catheterization, position of the head, Trendelenburg positioning, positive end expiratory pressure, Valsalva maneuver, and more recently, ultrasound guidance have been used [1,7,9].

Right IJV cannulation is usually preferred for several reasons, but rarely because the diameter and/or cross-sectional area of the right IJV are greater than those of the left. Before performing this study, a questionnaire was sent by e-mail to anesthesiologists and intensivists in 15 French university affiliated hospitals, in order to ascertain their IJV cannulation procedures. A total of 305 physicians replied (senior 77%, residents 23%). For the first attempt, 97% chose the right side, 2% chose the left, and 1% had no preference. The reasons for this choice (several items could be reported) were: direct straight access (n = 198), dominant hand side (n = 173), habit (n = 102), lower rate of complications (n = 23), but only 3% (n = 9) were aware of a difference in the diameter between the right and left IJV (unpublished data). Accordingly, it appeared that knowledge of the greater diameter of the right IJV in comparison with the left was not widespread, despite the fact that this anatomical characteristic has been reported previously.

TCE CT scans provide reliable data, allow neutral head positioning and analysis of eventual compressive factors or regional pathology, which could interfere with the measurements. Furthermore, TCE CT scans are not operator-dependent. We studied a general adult population, breathing spontaneously and who were normovolemic, and focused on the cross-sectional area (in addition to maximal diameter), which seemed to be more pertinent as regards central venous catheterization. Moreover, our measurements were automated, allowing precise evaluation of vessel dimensions.

The study was carried out at a time of controversy about the systematic use of 2D ultrasound guidance for central venous line placement. Real-time ultrasound guided cannulation reduces the failure rate, number of attempts, duration and cost of the procedure, and complications, especially for IJV catheterization [2]. Recently, guidelines have been published in order to promote this technique, but it is noteworthy that physicians were asked to retain their ability to use and teach the landmark method [10]. A previous study of ultrasound location of vessels followed by subsequent catheter placement with the landmark technique found no advantages over the standard landmark method [8]. Moreover, most practitioners consider ultrasound to be of little value when performing a common and simple procedure. Accordingly, it has been reported that 67% of cardiovascular anesthesiologists and critical care practitioners never or almost never use ultrasound to perform IJV catheterization, because they consider it unnecessary or because ultrasound is unavailable [11]. Before the generalization of ultrasound, our results suggest that right IJV must be catheterized at the first attempt. Ultrasound should be used in cases where the standard landmark method is difficult and/or hazardous (e.g. obese patients or other local anatomical variations).

## Conclusions

IJV anatomy with a greater diameter and cross-sectional area of the right IJV is an unknown reality. Left IJV puncture may therefore be more difficult or more dangerous in the case of a right dominant vein. Accordingly, the right IJV should be chosen for the first attempt.

## Key messages

• The right IJV has a greater diameter and cross-sectional area than the left one.

• In emergency and/or first attempt, the right IJV should be preferred than the left IJV.

## Abbreviations

CT: computed tomography; IJV: internal jugular vein; TCE: thoracic contrast-enhanced.

## Competing interests

The authors declare that they have no competing interests.

## Authors' contributions

DT made an important contribution to acquisition and data analysis. PS made a substantial contribution to the interpretation of data and wrote the final manuscript. CJ made a substantial contribution to the acquisition and analysis of data. BL made an important contribution to the statistical design and analysis. YM made an important contribution to the conception, interpretation of the data and the final manuscript.
